# The concurrent accuracy of the modified telephone interview for cognitive status and mini-mental state examination tools in detection of cognitive impairment among older adults

**DOI:** 10.1590/1980-5764-DN-2022-0005

**Published:** 2022-08-15

**Authors:** Delara Laghousi, Nayyereh Aminisani, Seyed Morteza Shamshirgaran, Ali Javadpour, Zahra Gholamnezhad, Neda Gilani, Mohammad Asghari-Jafarabadi, Fiona Alpass

**Affiliations:** 1 Tabriz University of Medical Sciences, Health Management and Safety Promotion Research Institute, Social Determinants of Health Research Center, Tabriz, Iran.; 2 Neyshabur University of Medical Sciences, Healthy Ageing Research Centre, Neyshabur, Iran.; 3 Shiraz University of Medical Sciences, Shiraz Geriatric Research Centre, Shiraz, Iran.; 4 Tabriz University of Medical Sciences, Faculty of Health, Department of Statistics and Epidemiology, Tabriz, Iran.; 5 Tabriz University of Medical Sciences, Faculty of Health, Road Traffic Injury Research Center, Department of Epidemiology and Biostatistics, Tabriz, Iran.; 6 Massey University, School of Psychology, Palmerston North, New Zealand.

**Keywords:** Interviews as Topic, Cognitive Dysfunction, Dementia, Psychological Tests, Aged, Iran, Entrevistas como Assunto, Disfunção Cognitiva, Demência, Testes Psicológicos, Idoso, Irã

## Abstract

Due to the need for face-to-face administration of many cognitive screening tests, it is not always feasible to screen large-scale samples.

**Objective:**

This study aimed to assess the discriminant validity of the Persian version of Telephone Interview for Cognitive Status (P-TICS-m) and Mini-Mental State Examination in the middle-aged Iranian population.

**Methods:**

The P-TICS-m and MMSE were administered to 210 randomly selected middle-aged community-dwelling adults who had been registered in the Neyshabur Longitudinal Study on Ageing. Participants also underwent psychological examination by two neurologists to assess cognitive impairment based on the *Diagnostic and Statistical Manual of Mental Disorders, Fifth Edition (DSM-V)* criteria. To evaluate the discriminant validity of P-TICS-m and MMSE with *DSM-V* criteria, the sensitivity, specificity, positive and negative predictive values (PPV and NPV), and positive and negative likelihood ratios (LR^+^ and LR^−^) were calculated.

**Results:**

The mean age of the participants was 59.6±6.8 years. The TICS and MMSE were highly correlated (r=0.635, p<0.001). The sensitivity, specificity, PPV, NPV, LR^+^, and LR^−^ to discriminate cognitive impairment were, respectively, 83%, 92%, 68%, 96%, 10, and 0.182 for MMSE and 100%, 13%, 19%, 100%, 1.16, and 0 for TICS-m. The receiver operating characteristic curve analysis results showed no statistically significant differences between P-TICS-m and MMSE.

**Conclusions:**

Our findings indicate that the TICS-m test can be used as a screening tool instead of the MMSE. Due to the low specificity and low PPV of the TICS-m compared to MMSE, the diagnosis should be confirmed using definitive diagnostic tests when a subject is classified as having cognitive impairment.

## RESUMO.

Diante da necessidade de administração face a face de muitos testes de triagem cognitiva, nem sempre é viável rastrear amostras em grande escala.

**Objetivo:**

O objetivo deste estudo foi avaliar a validade discriminante da versão persa do Telephone Interview for Cognitive Status (TICS-m) e do Miniexame do Estado Mental (MMSE) na população iraniana de meia-idade.

**Métodos:**

A versão persa do TICS-m (P-TICS-m) e do MMSE foi administrada a 210 adultos de meia-idade residentes na comunidade e selecionados aleatoriamente, que haviam sido registrados no Neyshabur Longitudinal Study on Ageing. Os participantes também foram submetidos a exame psicológico por dois neurologistas para serem avaliados quanto ao comprometimento cognitivo com base nos critérios do Manual de Diagnóstico e Estatística de Transtornos Mentais (DSM-V). Para avaliar a validade discriminante do P-TICS-m e do MMSE com os critérios do DSM-V, foram calculados a sensibilidade, a especificidade, os valores preditivos positivo e negativo (PPV e NPV) e a razão de verossimilhança positiva e negativa (LR+ e LR-).

**Resultados:**

A média de idade dos participantes foi de 59,6±6,8 anos. O TICS e o MMSE foram altamente correlacionados (r = 0,635, p <0,001). A sensibilidade, a especificidade, o PPV, o NPV, a LR+ e a LR- do MMSE para discriminar comprometimento cognitivo foram 83, 92, 68, 96%, 10, 0,182; e, para TICS-m, foram 100, 13, 19, 100%, 1,16 e zero, respectivamente. Os resultados da análise da curva característica de operação do receptor (ROC) não mostraram diferenças estatisticamente significativas entre P-TICS-m e MMSE.

**Conclusões:**

Nossos achados mostram que o teste TICS-m pode ser utilizado como ferramenta de triagem em vez do MEEM. Por causa da baixa especificidade e do baixo PPV do TICS-m em relação ao MMSE, o diagnóstico deve ser confirmado por meio de testes diagnósticos definitivos quando um indivíduo é classificado como portador de comprometimento cognitivo.

## INTRODUCTION

Dementia, a decline in memory and other cognitive functions, is a severe challenge for health care and social care systems^1^. According to the World Alzheimer’s Report, 47 million people live with dementia, and due to the aging of the population, its prevalence is expected to be triple by 2050^2^ ^,^ ^3^. In future, it is expected that Iran will encounter explosive growth in the number of older adults. The number of people aged 65 years and older is projected to rise from 5.7% in 2011 to 9.7% in 2030 and 25.2% by 2060. The current prevalence of dementia in Iran is 7.9% among individuals aged over 60 years and 13% among those aged over 80 years^4^. Despite the prevalence of Alzheimer’s disease (the most common cause of dementia), its diagnosis is often overlooked or mistaken^5^, and the rate of undetected dementia has been reported as high (61.7%)^6^. Early detection of Alzheimer’s disease provides opportunities for advanced care planning and improved prognosis^6^ ^,^ ^7^. Many cognitive screening instruments have been developed for the screening of cognitive impairment. Although the Mini-Mental State Examination (MMSE) has been used successfully to detect cognitive impairment, it is not always feasible to screen large-scale samples^8^ due to the need for face-to-face administration. In addition, due to a “ceiling effect” in mild cognitive impairments, its usefulness has been limited for research purposes. To overcome these limitations, several telephone interview-based cognitive screening instruments have been developed. One of the most popular instruments for this purpose is the Telephone Interview for Cognitive Status - modified (TICS-m), which correlates highly with the MMSE in Alzheimer’s disease^9^. The 13-item TICS-m is an abbreviated version of the original 21-item TICS-m and includes four cognitive domains, assigning the highest proportion of the total score to the memory component^10^. This study aimed to assess the accuracy of the Persian version of the 13-item TICS-m in comparison to the MMSE and *Diagnostic and Statistical Manual of Mental Disorders, Fifth Edition* (*DSM-V*) criteria in the detection of cognitive impairment among healthy people.

## METHODS

### Study population

This cross-sectional study was conducted in Neyshabur, Northeast of Iran, between January and March 2020. A total of 210 participants were recruited from community-dwelling adults aged 50 years and older who were registered with the Neyshabur Longitudinal Study on Ageing (NeLSA), which is an aging component of the Prospective Epidemiological Research Studies in Iran (PERSIAN)^11^. To decrease selection bias, random sampling was undertaken using a table of random numbers (the number of households with older adults) and samples were selected from the indwelling populations. Selected households were invited by phone to participate in the study. Adults aged 50 years and older who were willing to participate in the research and were able to read and write were included in the study. The exclusion criteria were as follows: adults with vision and hearing loss, use of hearing aids, having problems in the lower or upper limb that prevent walking or writing, history of psychological or neurologic disorders which cause cognitive impairment, intellectual or learning disabilities, brain surgery, alcoholism, drug abuse, head trauma with loss of consciousness for more than 2 h, and use of psychotropic drugs such as benzodiazepine, neuroleptic, antidepressant, anticonvulsant, and opioid within 7 days of cognitive evaluation.

### Procedure

#### Persian version of Telephone Interview for Cognitive Status - modified (P-TICS-m)

The P-TICS-m questionnaire, which was validated previously^12^, was applied in this study. First, all participants were screened using MMSE, and 4 weeks later, the P-TICS-m was administered by the same interviewer. All research assistants who administered the P-TICS-m and MMSE had master’s degrees in psychiatry and were specifically trained in the assessment procedure. The 13-item TICS-m questionnaire of Brandt et al. consists of six cognitive dimensions , namely, orientation (7 points), registration/free recall (10 points), attention/calculation (6 points), comprehension/semantic/recent memory (5 points), language/repetition (1 point), and delayed recall (10 points). In this questionnaire, the highest score is allocated to memory, which, unlike the MMSE test, gives 20% of its score to memory; in the TICS-m test, 56% of the total score is allocated to memory^9^. The total score ranges from 0 to 39. Individuals who scored ≤31 were considered having “mild cognitive impairment” and those who scored ≤27 were considered having “severe cognitive impairment”^13^.

#### Mini-Mental State Examination

The MMSE questionnaire includes five dimensions of cognition such as orientation (10 points), registration (3 points), attention and calculation (5 points), recall (3 points), and language (9 points). The total score ranges from 0 to 30. Individuals who scored <24 were considered having “mild cognitive impairment” and those who scored ≤17 were considered having “severe cognitive impairment”^14^ ^,^ ^15^ ^,^ ^16^ ^,^ ^17^.

#### Standard for comparison

Two psychiatric specialists examined all subjects who completed a neurological examination and administered the Short Test of Mental Status (STMS)^18^. The diagnosis of probable cognitive impairment was based on the *DSM-V* criteria^19^.

#### Statistical analysis

Numeric variables were expressed as mean and standard deviation, and categorical variables were expressed as frequency and percentage. The normality of data was examined using the Kolmogorov-Smirnov test. Due to the non-normal distribution of MMSE and TICS test scores, the Mann-Whitney U test and Kruskal-Wallis test were used to compare the two genders, age groups, and educational groups. The Spearman’s test was used to investigate the correlation between MMSE and TICS scores tests. To determine the accuracy of TICS-m and MMSE versus *DSM-V* criteria, sensitivity, specificity, positive and negative predictive values (PPV and NPV), and positive and negative likelihood ratios (LR^+^, LR−) were calculated along with their 95% confidence interval (95%CI). To compare the diagnostic accuracy of the TICS-m and MMSE tests, receiver operating characteristic (ROC) curve analysis was used to evaluate the significance of the difference between area under the curve (AUC) of TICS and MMSE tests versus *DSM-V* criteria, the Hanley and McNeil’s test^20^ was used. Youden’s index was also calculated to determine the best cutoff point for P-TICS-m with the highest sensitivity and specificity values in detecting patients with cognitive impairment. The data were analyzed using the IBM SPSS statistics software version 21 (IBM SPSS Statistics, Armonk, NY, USA) and the MS Excel 2013 software.

## RESULTS

### Descriptive results of P-TICS-m and MMSE questionnaires

The cognitive scores for the MMSE and P-TICS-m matched by gender, age, and education are displayed in Table 1. Out of 210 participants in the study, 108 (51.4%) were male, and 102 (48.6%) were female. The mean age of participants was 59.95±6.8 years (ranged from 50 to 87 years). The majority of participants (54%) were in the age group of 50-59 years.

**Table 1.** Distribution of median and interquartile range scores of Mini-Mental State Examination and Persian version of the Telephone Interview for Cognitive Status - modified by age, sex, and education (n=210).

Variables		n (%)	MMSE	P-TICS-m
			Median score (P_25_-P_75_)	Median score (P_25_-P_75_)
Gender	Male	108 (51.4)	27 (26-29)	29 (26-30)
	Female	102 (48.6)	25 (22.75-28)	27 (24-30)
Age (years)	50-59	114 (54.3)	27 (25-28.25)	28 (26-31)
	60-69	78 (37.1)	27 (23.75-28)	28 (25-29)
	≥70	18 (8.6)	24 (13.75-27)	21 (15.5-27.25)
Education level illiterate	Elementary	17 (8.1)	14 (13-20)	18 (13.50-20.5)
	Secondary	49 (23.3)	25 (23-28)	27 (24-29)
	Tertiary	24 (11.4)	27 (26-28.75)	28 (26-30)
	Diploma	4 (1.9)	24 (24-25.5)	24.5 (21.5-29)
	Academic	54 (25.7)	27 (25-29)	29 (26-30.25)
	Education	62 (29.5)	27 (26-29)	29 (27-31)

MMSE: Mini-Mental State Examination; P-TICS-m: Persian version of the Telephone Interview for Cognitive Status - modified.

### Correlation between TICS and MMSE tests

Spearman’s test was used to examine the correlation between TICS and MMSE tests. Despite the different scoring ranges of both tests (0-30 for the MMSE test and 0-39 for the TICS test), there was a strong, direct, and significant correlation between the scores of both tests (r=0.635, p<0.001).

### Concurrent validity of P-TICS-m with MMSE (As a most commonly used screening test) in the detection of cognitive impairment

The sensitivity, specificity, PPV, and NPV of P-TICS-m compared with MMSE were 100%, 14%, 23%, and 100%, respectively (Table 2). According to the results of ROC analysis, the AUC of the P-TICS-m was 0.88 (95%CI 0.83-0.93, p<0.0001). This AUC indicates that P-TICS-m has a good performance in identifying cognitive impairment subjects from healthy ones compared to MMSE (Table 2).

**Table 2.** Accuracy of the Persian version of the Telephone Interview for Cognitive Status - modified versus MMSE test in identifying cognitive impairment from healthy older adult (n=210).

	Estimate	95%CI
Sensitivity	1	0.92-1
specificity	0.145	0.099-0.206
PPV	0.237	0.181-0.303
NPV	1	0.862-1
LR^+^	1.17	1.098-1.244
LR^−^	0	0 to 0
AUC	0.888	0.837-0.938

PPV: positive predictive value; NPV: negative predictive value; LR^+^: positive likelihood ratio; LR^−^: negative likelihood ratio; AUC: area under the curve.

### Discriminant accuracy of P-TICS-m and MMSE for cognitive impairment versus DSM-V criteria (as a standard test)

#### Having cognitive impairment or not

**Figure 1.**
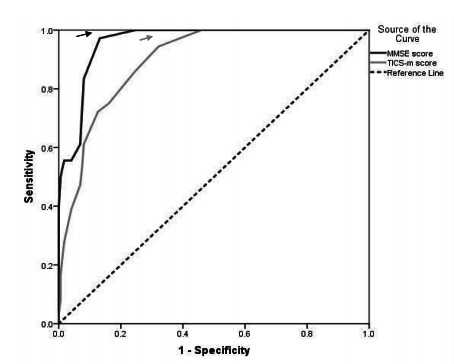
Receiver operating characteristics of the TICS-m and MMSE instruments.

MMSE: Mini-Mental State Examination; P-TICS-m: Persian version of the Telephone Interview for Cognitive Status - modified.

The sensitivity, specificity, PPV, and NPV of the P-TICS-m using *DSM-V* criteria were 100%, 13%, 19%, and 100%, respectively. The sensitivity, specificity, PPV, and NPV of the MMSE test using *DSM-V* criteria were 83, 92, 68, and 96%, respectively (Table 3). Also, Table 3 shows the results of the ROC curve analysis for the assessment of the discriminant validity of the P-TICS and MMSE. The AUC of MMSE was higher than the P-TICS-m (0.959 vs. 0.896), but there was no significant difference between the P-TICS-m and MMSE (difference of both AUC=0.06, p=0.188073) (Table 3 and Figure 1). The P-TICS-m had 94.4% sensitivity and 67.8% specificity at the optimal cutoff score of ≤27.5, and the MMSE showed 97.2% sensitivity and 86.8% specificity at the optimal cutoff score of ≤24.5 (Table 3).

**Table 3.** Discriminant accuracy of Telephone Interview for Cognitive Status - modified and Mini-Mental State Examination questionnaire for cognitive impairment using Diagnostic and Statistical Manual of Mental Disorders-V criteria.

Diagnostic test characteristics	MMSE		TICS-m	
	Estimate	95%CI	Estimate	95%CI
Sensitivity	0.833	0.681-0.921	1	0.904 to 1
specificity	0.92	0.869-0.951	0.138	0.094-0.197
PPV	0.682	0.534-0.8	0.194	0.143-0.256
NPV	0.964	0.923-0.983	1	0.862-1
LR+	10.413	6.138-17.475	1.16	1.093-1.231
LR^−^	0.182	0.087-0.377	0	0-0
AUC	0.959	0.935-0.983	0.896	0.850-0.942
Cutoff point; sensitivity/specificity	24.5; 0.972/0.868		27.5; 0.944/0.678	

MMSE: Mini-Mental State Examination; TICS-M: Modified Telephone Interview for Cognitive Status; PPV; positive predictive value; NPV: negative predictive value; LR+: positive likelihood ratio; LR^−^: negative likelihood ratio; AUC: area under the curve.

#### Detection of mild and severe cognitive impairment from those without cognitive impairment

The sensitivity of P-TICS-m in identifying those with severe and mild cognitive impairment was 100% and 9.5%, and its PPV was 16% and 0%, respectively. The sensitivity of the MMSE in the detection of those with severe and mild cognitive impairment was 86% and 71%, and its PPV was 100% and 48%, respectively (Table 4).

**Table 4.** Discriminant accuracy of Telephone Interview for Cognitive Status - modified and Mini-Mental State Examination questionnaire for mild and severe cognitive impairment from those without cognitive impairment using Diagnostic and Statistical Manual of Mental Disorders-V criteria.

Diagnostic test characteristics	MMSE		TICS-m	
	Severe cognitive impairment	Mild cognitive impairment	Severe cognitive impairment	Mild cognitive impairment
Sensitivity	0.867	0.714	1	0.095
PPV	1	0.484	0.167	0.0
LR+	10.837	8.925	1.160	1.102
LR^−^	0.144	0.310	0	0.362

MMSE: Mini-Mental State Examination; TICS-M: Modified Telephone Interview for Cognitive Status; PPV; positive predictive value; LR+: positive likelihood ratio; LR^−^: negative likelihood ratio.

## DISCUSSION

The present study showed a signiﬁcant correlation between the P-TICS-m and the widely used cognitive function test of MMSE. The original version of the TICS test also correlates very highly with the MMSE in Alzheimer’s disease^9^ ^,^ ^21^. However, in the study by de Jager et al., the correlation was relatively low^10^. In terms of discriminant validity of the P-TICS-m compared with MMSE in detection of subjects with cognitive impairment from subjects without cognitive impairment, results showed that the sensitivity, specificity, PPV, and NPV of P-TICS-m were 100, 14, 23, and 100%, respectively. These results indicate that the P-TICS-m can detect all the MMSE diagnoses as having cognitive impairment. In addition, when a subject is diagnosed as healthy using the P-TICS-m, it is 100% probable that the same subject will be assessed as healthy using the MMSE. However, the P-TICS-m classifies many participants as having a cognitive impairment that the MMSE classifies as healthy. The MMSE test is not the gold standard test for diagnosing cognitive impairment and thus may not provide an accurate assessment of the TICS-m test^22^. For more specific conclusions about the discriminant validity of these tests, neuropsychological evaluation by two neurologists and diagnosis based on *DSM-V* criteria were used. According to our results, the sensitivity and NPV of the P-TICS-m using *DSM-V* criteria were high (100%), but its specificity and PPV were low (13 and 19%, respectively). Also, its LR^+^ ratio was low (1.16), which means that the probability of over-diagnosis of the P-TICS-m is high and that 80% of healthy subjects are mistakenly classified as cognitive impaired (FP=80%). Therefore, the probability of further follow-ups will increase. However, the P-TICS-m correctly rules out cognitive impairment. The predictive value of a test is not just a test property and is influenced by prevalence and the setting in which the test is used. When the test is applied in a specialist setting such as a cognitive disorder clinic, it will have a higher predictive value than when the test is applied in non-specialist settings, such as community or primary care. In other words, the interpretation of a positive or negative diagnostic test result varies from setting to setting, according to the prevalence of disease in the particular setting. In these cases, it is recommended to use LR^+^ and LR^−^ ^22^. In this study, the LR^−^ of both TICS-m and MMSE was very low. Therefore, the probability of a false-negative test result to the possibility of a true negative test result^23^ is very low. This means that these tests do not misdiagnose healthy people. According to our results, the diagnostic accuracy of MMSE using *DSM-V* criteria was better than TICS-m, in particular its specificity, PPV, and LR^+^ (LR^+^=10.83). That is, the chance of true positive test results to false-positive test results is 10 times.

In addition, out of the three positive results of the MMSE in suspected participants, two subjects were correctly classified as having a cognitive impairment (PPV=68%). The results of a meta-analysis that evaluated the accuracy of the MMSE indicated that the accuracy of a diagnostic test varies with the context in which it is used. For example, in clinics or specialized hospitals, the PPV of the MMSE was high, but the NPV of the MMSE in these settings was moderate. Conversely, in a community or primary care setting, the NPV of the MMSE was high, and the PPV of the MMSE was low. The results of our study were also in line with the findings of this study. Therefore, it is suggested that the MMSE be used for ruling out dementia in the community or primary care settings, but to confirm the diagnosis of dementia, other definitive diagnostic tests should be used^24^. Comparing the accuracy of P-TICS and MMSE using ROC curve analysis, our findings revealed that although the AUC of the MMSE was higher than TICS-m, there was no statistically significant difference between the two tests. However, given that the AUC of the MMSE is slightly higher than the TICS-m, it can be concluded that the MMSE works better^25^ ^,^ ^26^.

Considering the high NPV and the low LR^−^ of the TICS-m compared to the MMSE, there is no need for confirmatory tests when a person is classified as healthy. However, due to the low specificity and low PPV of the TICS-m compared to MMSE, the probability of false positive increases. Therefore, when a person is classified as cognitive impaired, the diagnosis should be confirmed using definitive diagnostic tests.
